# Conducting retrospective impact analysis to inform a medical research charity’s funding strategies: the case of Asthma UK

**DOI:** 10.1186/1710-1492-9-17

**Published:** 2013-05-07

**Authors:** Stephen R Hanney, Amanda Watt, Teresa H Jones, Leanne Metcalf

**Affiliations:** 1 Health Economics Research Group, Brunel University, Uxbridge UB8 3PH, UK; 2 RAND Europe, Westbrook Centre, Milton Road, Cambridge, CB4 1YG, UK; 3 Asthma UK, Summit House, 70 Wilson Street, London, EC2A 2DB, UK

**Keywords:** Asthma, Asthma UK, Research impacts, Societal impacts, Clinical guidelines, University spin-out companies, Product development, Immunotherapy, Payback Framework, Research funding strategy

## Abstract

**Background:**

Debate is intensifying about how to assess the full range of impacts from medical research. Complexity increases when assessing the diverse funding streams of funders such as Asthma UK, a charitable patient organisation supporting medical research to benefit people with asthma. This paper aims to describe the various impacts identified from a range of Asthma UK research, and explore how Asthma UK utilised the characteristics of successful funding approaches to inform future research strategies.

**Methods:**

We adapted the Payback Framework, using it both in a survey and to help structure interviews, documentary analysis, and case studies. We sent surveys to 153 lead researchers of projects, plus 10 past research fellows, and also conducted 14 detailed case studies. These covered nine projects and two fellowships, in addition to the innovative case studies on the professorial chairs (funded since 1988) and the MRC-Asthma UK Centre in Allergic Mechanisms of Asthma (the ‘Centre’) which together facilitated a comprehensive analysis of the whole funding portfolio. We organised each case study to capture whatever academic and wider societal impacts (or payback) might have arisen given the diverse timescales, size of funding involved, and extent to which Asthma UK funding contributed to the impacts.

**Results:**

Projects recorded an average of four peer-reviewed journal articles. Together the chairs reported over 500 papers. All streams of funding attracted follow-on funding. Each of the various categories of societal impacts arose from only a minority of individual projects and fellowships. Some of the research portfolio is influencing asthma-related clinical guidelines, and some contributing to product development. The latter includes potentially major breakthroughs in asthma therapies (in immunotherapy, and new inhaled drugs) trialled by university spin-out companies. Such research-informed guidelines and medicines can, in turn, contribute to health improvements. The role of the chairs and the pioneering collaborative Centre is shown as being particularly important.

**Conclusions:**

We systematically demonstrate that all types of Asthma UK’s research funding assessed are making impacts at different levels, but the main societal impacts from projects and fellowships come from a minority of those funded. Asthma UK used the study’s findings, especially in relation to the Centre, to inform research funding strategies to promote the achievement of impact.

## Background

Globally research funders are under growing pressure to demonstrate the returns or impacts that arise from their research funding [1-4]. In 2006, the UK Evaluation Forum, which brought together the Academy of Medical Sciences, the Medical Research Council (MRC) and the Wellcome Trust, considered ways of assessing the benefits of medical research and called for further studies [2].

For charitable patient organisations that fund medical research it is increasingly important to demonstrate that the money they have invested is leading to improvements in the healthcare and quality of life of the patients they exist to support [5]. Charities are accountable for this in a formal way (for example, to regulators, funders or members) and in a moral way (for example, to beneficiaries, service users, partner charities, staff, volunteers and the general public).

Asthma UK (under its current and previous names) has been funding research since 1927. Since the formation of the National Asthma Campaign in 1989, the charity has spent over £50 million on research to understand more about asthma, its causes and treatments. It has been spending up to £3 million and funding between ten and twenty new projects each year. Asthma UK has historically provided various types of funding for research, including long-term support for two professorial chairs over the last twenty years, medium-term support through a research fellowship scheme, and project support through an annual grant round. Since 2005 the charity has jointly funded the pioneering collaborative MRC-Asthma UK Centre in Allergic Mechanisms of Asthma (the ‘Centre’) based at both King’s College London and Imperial College London. Asthma UK has more recently introduced funding for PhD studentships, initially linked to the Centre.

Asthma UK has a relatively limited budget and so has long engaged with the scientific community to help identify the areas of research that would be most likely to achieve its objectives [6]. However, in response to the charity’s wider range of accountabilities, Asthma UK decided to review various aspects of its role as a research funder. These included, firstly, increasingly ensuring people affected with asthma were meaningfully involved in reviewing all proposals for funding, and, secondly, building greater understanding of what had been achieved through the breadth of its previous research funding activities, so as to help inform an enhanced research strategy. This article focuses primarily on the latter, but provides some evidence related to the former.

Although the importance of charities being able to demonstrate the impact of their research funding is widely recognised, attempts to assess rigorously the impact of research funding have been limited, and not just for charitable research funders. This is largely due to the huge difficulties of demonstrating impact in research, attributable to the fact that many pieces of research might contribute to achieving some impacts, and a huge time lag can be involved before tangible benefits are realised [7].

Of the relatively small number of impact studies reported in the literature, the Payback Framework developed by the Health Economics Research Group (HERG) is described as the approach used most often [8-10]. It constitutes a framework for addressing the conceptual issues, and collecting, analysing and reporting data in a reasonably consistent manner to capture the impacts and outputs of research [8]. As such, the Payback Framework has been used as a tool to help funders and stakeholders in research to think about what the likely impacts from research can or might be.

For a research funder there could be clear benefits in having an assessment made of their full portfolio of research funding. Yet, attempts to assess the impact of a wide-ranging research funder’s full portfolio of research are even rarer than assessment of selected case studies or specific programmes. This is because they require the combination of two different approaches:

• achieving breadth of coverage to give a reasonable picture of the impacts from the full portfolio;

• conducting the detailed analyses needed to address issues such as how far impacts can be attributed to the specific research being examined.

Doing this rigorously requires the research instruments (surveys and case studies adopting triangulation techniques etc.) to be able to tackle issues such as responders’ bias and selective recall.

In 2008, Asthma UK approached HERG to apply their Payback Framework to identify the benefits that have arisen from the charity’s various forms of research support and thereby help Asthma UK to continue to use its funds to best effect in terms of maximising benefits for people with asthma in its future research strategy. The team from HERG and RAND Europe conducting the independent retrospective impact analysis provided a complete report to Asthma UK, and an additional 300 page volume of supporting case studies.^a^

The objectives of this article are to describe the methods and results of this evaluation of the impacts from Asthma UK’s research (including how the difficulties in assessing the range of impacts from such a comprehensive portfolio were addressed), and then to discuss how the findings have been used by Asthma UK to inform its research funding strategy.

## Methods

Assessing the impacts of the full portfolio of a health research funder requires a combination of methods. Those applied in this study included a review of the data already gathered by Asthma UK, a survey sent to the researchers, and detailed case studies. However, such methods are enhanced when informed by a conceptual framework that can help organise the data collection, analysis and reporting in as consistent a manner as possible [8], in contrast to being a collection of one-off case studies.

**Figure 1:**
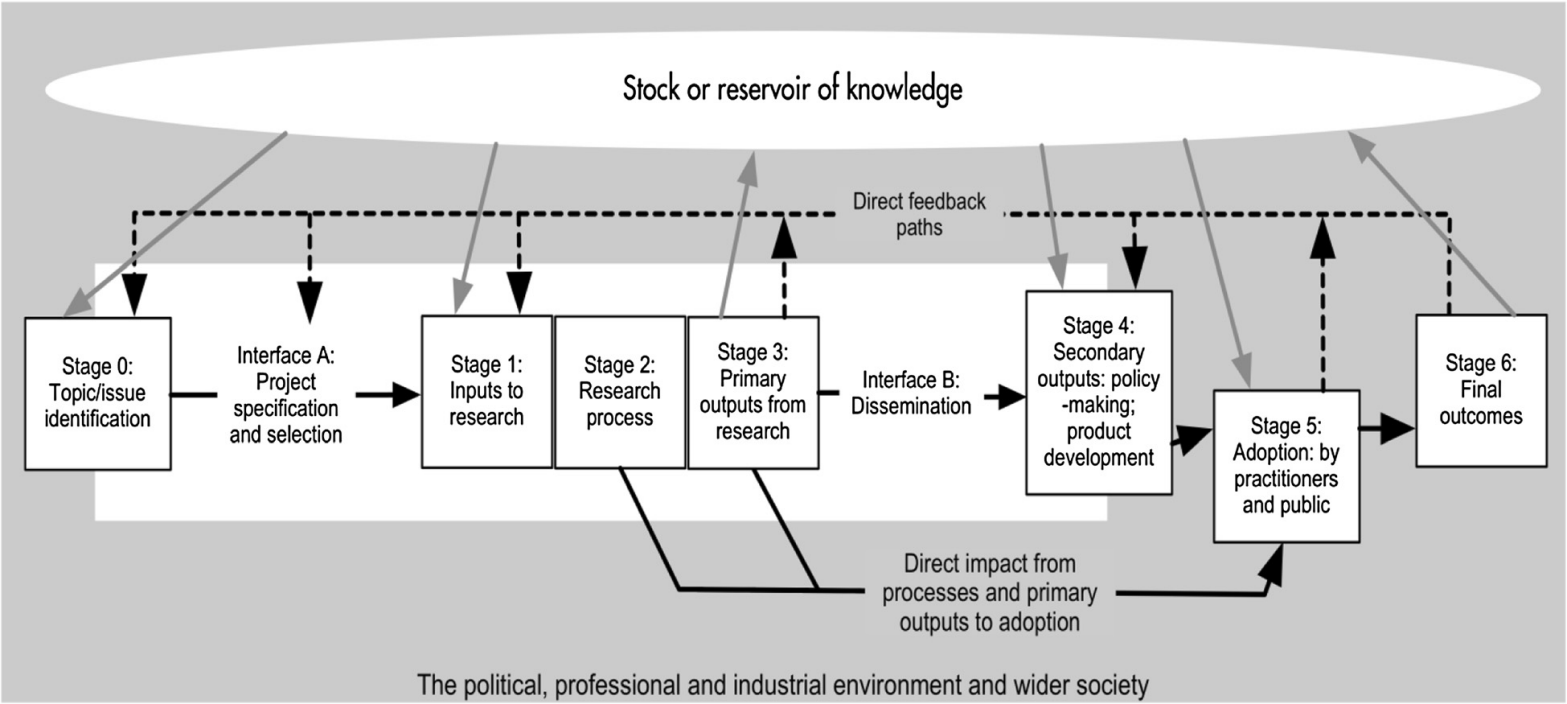
**The logic model of HERG’s Payback Framework used to organise the assessment of research impact. **Source: Adapted from Hanney et al., 2004 [5].

We therefore organised this project around an adaptation of the HERG Payback Framework [5,11]. The framework combines two aspects: a multi-dimensional categorisation of benefits from health research and the payback logic model. The multi-dimensional categorisation of benefits includes traditional categories such as the knowledge production represented by publications, research capacity building and the targeting of future research. But it also incorporates wider impacts that are increasingly viewed as important by research funders, especially charitable patient organisations, including: informing policy and product development (which includes clinical policies such as guidelines); health and health sector benefits; and broader economic benefits. The logic model is shown in Figure 1 and presents a simplified version of the processes involved in commissioning and undertaking the research, and in generating the full range of impacts. It helps identify where the various categories of impacts themselves might arise, but recognises there is often considerable feedback between the different stages.

Pursuing the analysis through the various stages of the logic model helps address attribution issues. The early stages of the model focus on the context in which the research was undertaken, and the motivations behind it. Analysis here increases opportunities for identifying impacts in the later payback categories, and attributing them (at least partially) to the specific research funded. Similarly, analysis at the interfaces between researchers and research users can be important in understanding how far impacts have been achieved. Previous studies provide fuller accounts of the Payback Framework and how it is applied to inform the methods used for data collection, analysis and reporting when the focus has been on selected case studies or on specific programmes [5,8,11-13]. The study received appropriate ethical approval from the University Research Ethics Committee at Brunel University.

### Survey

We adapted a survey used previously to examine the impacts from the research funded by the Health Technology Assessment programme in England [8], but amended it to increase its relevance to Asthma UK. The full survey can be seen as an additional file [see Additional file 1]. For each project we identified publications already on Asthma UK’s database and inserted them into the relevant survey. Respondents were then asked to make any necessary amendments. Many of the survey questions not only asked about whether certain developments had occurred (for example, whether the research led to any follow-on projects) but also asked about the degree of influence on such decisions made by the original research funded by the charity. This was in recognition of the fact that just because Asthma UK funding had been used to create scientific evidence, it did not guarantee that impact had been achieved in a simple and linear manner, and it was desirable to attempt to identify the level of the charity’s contribution.

We also wanted to make some calculation of the amount of follow-on funding that might reasonably be thought to have come from the original projects. Therefore, we not only asked grant-holders to record the funder of the follow-on research and the amount, but also asked the responders to use one of three categories (considerable/moderate/small) to describe the contribution to securing or informing the follow-on funding made by the original Asthma UK funding.

We piloted the survey before sending it to all 153 lead researchers (or principal investigators – PIs) awarded project grants from 1996 (a date from which Asthma UK held reasonable archival data) that had been completed by 2006, and to the ten researchers whose fellowships had been completed. We recorded the survey responses in an Access database, and developed innovative analysis techniques for various issues. For example, previous studies had just totalled the recorded amount of follow-on funding but this was criticised as exaggerating the role of just one funder when several should probably share the credit. Therefore, we not only asked PIs to make an estimate of the contribution to the follow-on funding made by the original Asthma UK-funded research, but also made a best estimate of what the categories ‘considerable’, ‘moderate’ and ‘small’ might mean in quantitative terms. We took the amount of follow-on funding recorded for projects and multiplied it by the following proportions: considerable: 0.9; moderate: 0.5; small: 0.1. This gave us a total of adjusted follow-on funding for all the projects for which a survey had been completed. We then calculated the total funding supplied by Asthma UK for all the projects that completed a survey, irrespective of whether or not the PI reported any follow-on funding. Finally we calculated the ratio of total follow-on funding (adjusted for the contributions made and excluding the follow-on funding that came from Asthma UK itself) to original Asthma UK funding.

### Case studies

The project’s research team, in conjunction with the project’s Advisory Committee, used information obtained from the survey about claims for impact in the various categories to inform the selection of nine projects and two fellowships on which to undertake case studies – these are listed on Table 1. We also wanted to conduct case studies on the work of the two Asthma UK professorial chairs who had been funded since 1988, and had, therefore been a key element in Asthma UK’s funding strategy, and on the MRC-Asthma UK Centre which was a major recent development.

**Table 1:** Case studies on nine project grants and two fellowships

**Project grants**		
**Researcher: title and location at time of proposal**	**Title of research (and ID)**	**Duration in months**
**Bradding**, Dr. Peter. Glenfield Hospital, University of Leicester	Human mast cell adhesion to bronchial epithelium and airway smooth muscle (02/014)	24 (2003–05)
**Britton**, Professor John. University of Nottingham	Study of the role of parasites, dust mite exposure and other environmental factors in the aetiology of asthma and atopy in urban and rural Ethiopia (98/014)	12 (1998–00)
**Bush**, Dr. Andrew. Imperial College, London	Pathology of severe asthma in children (01/037)	23 (2001–03)
**Durham**, Professor Stephen. Imperial College, London	Influence of grass pollen immunotherapy (IT) on allergen-specific peripheral blood T-cell lines: allergy or immune deviation? (97/069)	24 (1997–99)
**Hawrylowicz**, Dr. Catherine. King’s College, London	IL-10: A critical regulator of inflammation and glucocorticoid responsiveness in asthma (00/023)	36 (2000–03)
**Hubbard**, Dr. Richard. University of Nottingham	A birth cohort study of the impact of asthma, acute exacerbations of asthma and asthma therapies on the risk of pregnancy complications and adverse perinatal and paediatric outcomes (04/019)	24 (2004–06)
**Johnston**, Dr. Sebastian. University of Southampton	Rhinovirus-induced regulation of adhesion molecule expression in asthma exacerbations (332)	24 (1996–98)
**Pavord**, Professor Ian. Glenfield Hospital, University of Leicester	The immunopathology and corticosteroid responsiveness of non-eosinophilic asthma (02/036)	24 (2002–05)
**Sutton**, Professor Brian. King’s College, London	Structure based design of inhibitors of IgE binding to the mast cell receptor (97/033)	36 (1997–01)
**Fellowships**		
**Custovic**, Dr. Adnan. University of Manchester	Gene-environment interaction in the development of atopy, asthma and other allergic diseases (RF01C)	60 (2000–04)
**Thomas**, Dr Mike. University of Aberdeen	Primary care asthma management: diagnosis, assessments and effective therapy (RF09T)	60 (2005–10)

In total, therefore, we undertook 14 case studies using archival and documentary review, interviews and bibliometric analysis. There is inevitably a bias introduced into the study by concentrating on case studies that are thought likely to be positive examples. However, where the main purpose of the case studies is to inform long-term strategy through a richer analysis of the range of impacts that arise - and how they manifest in different payback categories over time - such cases need to have sufficient content with which to explore what has happened to Asthma UK-funded research.

For the project and fellowship case studies we interviewed the PIs and also, where possible, one or two further members of the relevant research team. As in previous studies, we used a semi-structured interview schedule informed by the Payback Framework. For all the case studies we adopted a ‘rolling triangulation’ approach [14] in which we used the data gathered from documentary and archival review, plus that from earlier interviews, to inform the creation of a specific schedule for each interview [see Additional file 2]. The Payback Framework provided a conceptual framework to organise not only the data collection but also the analysis of the data and its presentation in each case study.

For the case studies on the two professorial chairs and the Centre, the Payback Framework again informed the interview schedules and write-up. In these cases, however, we applied it in an innovative way to take account of the breadth of topics covered, and to allow analysis of how the nature of the funding (long-term in the case of the chairs, and collaborative in the case of the Centre) might facilitate the production of a range of impacts. Various interviews were conducted for each of these three case studies. In total, 15 interviewees helped inform the Centre case study. They were: the two professorial chairs, who became respectively Director and Deputy Director of the Centre upon its creation in 2005; nine other members of the Centre; and four independent experts.

As part of the case study analysis we searched major national and international clinical guidelines related to asthma^b^ to see how far the Asthma UK-funded research had influenced the clinical guidelines, which are regarded as a form of clinical policy [14]. To obtain a fuller idea of the contribution made by the Asthma UK-funded research we took the analysis further than in previous studies and on this occasion examined the number of times the relevant articles were cited in the guideline, the importance of specific points being supported by Asthma UK-funded research, and how far the Asthma UK-funded paper was the only, or key, evidence supporting the relevant points. We also used a triangulation approach as far as possible to inform the write-up of the case studies. A starting point for each case study was the survey, but the archival and documentary review and the interviews allowed claims made in the survey to be checked and the issues further explored.

Case studies such as these on specific pieces of research refer explicitly, and unavoidably, to the work of identified individuals and teams. We therefore obtained clearance for the text of each case study from the PI of the project (fellowship or chair), and also from any other interviewee whose interview evidence is used in an identifiable manner. This is not just a matter of courtesy, but is an important aspect of ensuring the scientific quality of the reported findings and ethical conduct of the study.

### Analysis of use of the report’s findings

The report was considered by Asthma UK as part of its review of its research strategy. For this article the way in which the report has been drawn upon by Asthma UK was analysed by LM, using her position from within the organisation.

## Results

Ninety six surveys were returned (90 projects and six fellowships) giving a response of 59% and we successfully completed the full complement of 14 case studies. The findings of both the survey and case studies are presented according to the categories of impact from the Payback Framework, and then briefly summarised to demonstrate the impact from each type of funding.

### Impacts relating to each impact category

#### Knowledge production

The 90 projects recorded an average of four peer-reviewed journal articles per project, but four of these projects did not record producing any articles. There is a possible bias in the results as there is some evidence from Asthma UK’s database of publications, as described in the full report (see End Note^a^), showing that the average number of publications already known to Asthma UK is slightly higher for the projects on which surveys were returned, than for those that were not. The research fellows who completed the survey recorded an average figure of 11 peer-reviewed articles. This higher average figure reflects the view of some fellows that because their career at that time was being funded by Asthma UK, all, or at least most, of their resulting publications could be counted as having some link with the fellowship. Since their appointments in 1988, the two Asthma UK professorial chairs, Tak Lee at King’s College London, and Tim Williams, at Imperial College London, published a total of over 500 publications.

For example, Tim Williams, conducted a major stream of research in the search for the chemoattractants for eosinophils, a type of white blood cell regarded as being as of considerable importance. Williams and his colleagues identified a potent endogenous chemoattractant with high specificity for eosinophils. Williams named this chemokine Eotaxin; the main paper describing it was published in the *Journal of Experimental Medicine* (Jose, 1994) [15] and has been cited over 600 times. It has helped target considerable further research including by Williams and his colleagues. They also successfully sought patents on their work that they have been granted worldwide on Eotaxin and antibodies to it. Further examples of key publications from Asthma UK-funded chairs, projects and fellowships [16-18] are described in the case studies summarized in Additional files 3, 4 and 5.

#### Research training and capacity building

The 90 PIs who completed surveys claimed that at least 62 higher degrees have been obtained or were expected at least in part as a result of Asthma UK’s project funding. An additional 15 are linked to the six fellowships covered in this analysis. These 77 higher degrees include 45 PhDs and 21 MDs. Asthma UK has now funded PhD studentships at the MRC-Asthma UK Centre (and several elsewhere jointly with the MRC). According to the case study analysis, this is making an important contribution to the substantial advances in research training in asthma coming from the Centre.

In addition, the researchers from 64% of the funded projects that participated in the survey reported details of career development for at least one team member as a result of the Asthma UK project funding. This included assisting promotion for PIs, and also project researchers moving forward to gain fellowships from major funders and continue their research in the asthma field.

#### Targeting further research and attracting further income for asthma research

In total Asthma UK invested some £9.2 million in the 90 projects included in the analysis. The PIs claimed that such investment helped to target, i.e. identify relevant research questions for, 99 follow-on projects conducted by themselves or members of their team. These follow-on projects received almost £25 million in funding from funders other than Asthma UK. However in the survey, the PIs indicated that the intellectual contribution from the original Asthma UK project to some of the largest follow-on grants was often only moderate, or sometimes small. Taking this into account, in the manner described above, the £25 million of total follow-on funding could, at a best estimate, be counted as equivalent to £12.9 million follow-on funding linked to Asthma UK’s £9.2 million original investment, So, every pound invested by Asthma UK in the research projects funded between 1996 and 2006 is likely to have attracted at least £1.40 in follow-on research funding from sources other than Asthma UK. In addition, in at least 35 cases, other researchers were reported to have built on the findings from the Asthma UK funded projects.

In some instances Asthma UK funding played a ‘pump-priming’ role, enabling the researcher to leverage larger sums from other health research funding bodies, a major example of which is described in the case study on peptide-based immunotherapy research [18] [See Additional file 5]. On occasions where Asthma UK itself funded the follow-on research this has, at least in retrospect, amounted to a programme of work according to survey and case study data. Sometimes these major streams of work, involving a succession of project grants from Asthma UK undertaken over many years (including some dating back before 1996), have been important in helping to move towards the wider impacts (policy or product development, and health gains), often supplemented by funding from other sources. Examples include the research that started with an Asthma UK-funded project on IL-10 and glucocorticoid responsiveness in asthma which was outlined on Asthma UK’s web site as follows: ‘A series of Asthma UK funded projects led by Professor Catherine Hawrylowicz in Professor Lee’s department has attracted particular interest, as the work has resulted in a clinical trial to explore whether vitamin D can improve the effectiveness of steroid treatments for asthma in those who are normally resistant to their benefits’ [19].

In a similar manner, the chairs directly brought in additional funding for asthma research. Since 1995, Tak Lee successfully applied for grants from the MRC, Wellcome Trust and NHS R&D Programme alone worth about £6 million, in addition to the £2.7 million from the MRC to the MRC-Asthma UK Centre. King’s College also made considerable infrastructural investment into Lee’s department and the Centre. Overall Lee built up a substantial research division. Similarly, Tim Williams secured three major programme grants from the Wellcome Trust to support his streams of asthma related research and has secured university funding for various posts. Those interviewed for the case study consistently claimed that the creation of the MRC-Asthma UK Centre has helped secure additional funding for asthma research. This is both in terms of the core funding provided by the MRC for additional facilities at the Centre, and the various other ways in which PIs at the Centre are successfully applying for funding (including through Asthma UK), partly by demonstrating the strength of the environment within which the research would be undertaken.

#### Informing policy development

In the survey just 13% of projects claimed to have made an impact on policy already, and 17% expected such an impact in the future. As part of the triangulation process, checks were made of the impacts on guidelines claimed in surveys by PIs of projects on which case studies were later conducted. Generally exaggerated claims had not been made. Indeed, the case studies often identified additional examples of the research having been cited on guidelines. On the basis of this analysis, we broadly accepted the claims made in the surveys on which case studies were not conducted.

According to the survey and case studies, three of the six fellows claimed already to have made an impact on policies and/or guidelines - longer-term salary funding can allow fellows to develop a distinct role or focus which lends itself more to policy development. For example, a key paper by Mike Thomas [20] is already being used in recent guidelines from the British Thoracic Society/Association of Chartered Physiotherapists in Respiratory Care [21] to support the use of breathing exercises for improved control of asthma and quality of life.

Using the methods described above we were able to establish that not only were papers from some Asthma UK-funded research cited on international and national guidelines, and other policy statements, but we also identified some examples when they supported key points and were either the only evidence used to support the point, or an important part of it. Some examples are given in the case study on research on immunotherapy that was part funded by Asthma UK [22,23] [see Additional file 4]. Other examples of national and/or international guidelines (or specific sections) influenced by Asthma UK-funded research, along with the Asthma UK-funded paper cited in the guideline, include ones on: cough [24,25]; asthma diagnosis in children [26,27]; and inhaled corticosteroid resistance [26,28].

Whilst there are often time lags involved in achieving an impact on policy, Asthma UK also supported some explicit and successful attempts to provide evidence for guidelines in areas in which there were gaps. In such situations there could be a rapid uptake of the findings. For example, Richard Hubbard specifically applied to Asthma UK for funding to develop stronger evidence on the safety of asthma medicines during pregnancy, an area where expert analysis of existing guidelines indicated the evidence was weak [29]. The findings from this project were published in late 2008 [30], and were almost immediately incorporated into an update of the 2008 guidelines from the British Thoracic Society and Scottish Intercollegiate Guidelines Network published in June 2009 [31].

#### Informing product development

In the survey only a small minority of projects – just 17% - included in this research claim an impact on product development already, and 31% claim to expect some future impact. The impact on product development from Asthma UK projects and from chair funding takes various forms, including helping to identify new roles for existing products, contributing to the evidence base for the development and application of major new drugs, and contributing to new therapies being developed by university spin-off companies.

In the stream of Asthma UK-funded projects on IL-10 and glucocorticoid responsiveness in asthma described above, the important new therapy being tested – vitamin D for steroid resistant asthma [32] – might involve using existing products in a new way. The background and significance of a stream of work in supporting the development of anti-leukotriene medicines [16,33,34] is explained in more detail in an additional file taken from the case study on Tak Lee’s work as an Asthma UK professorial chair [see Additional file 3].

Based on the projects included in this analysis, Asthma UK-funded research has also contributed to product development now being trialled by university spin-out companies founded by the researchers in several cases. The stream of research that showed T cell peptides have potential in the treatment of cat allergies led to the establishment of Circassia, an Imperial College London spin-out company. The progress made, including successful phase II trials, is described in an additional file which provides further details about the research and contains updates on the important findings published from a subsequent joint Canadian/UK study that is continuing this stream of work [see Additional file 5].

Many years of research by Stephen Holgate and colleagues led to the ideas behind the development of Interferon-beta treatment for rhinoviruses (common cold infections) that cause many asthma exacerbations. At a crucial time Asthma UK provided project funding for Holgate that contributed to key advances, although the MRC had supported much of the stream of work [35]. The spin-out company, Synairgen, successfully completed Phase 1 trials of the treatment and started Phase II in March 2010 [36]. The significant further progress made after the formal end of the retrospective impact analysis is described in the Discussion.

#### Health gain and broader economic benefits

Of the funded projects included in this research, again only a small minority (10%) claim to have already made an impact in any of the various forms this could take, with 6% of projects believing they had made an impact specifically in relation to health gains. Thirty-one percent of projects suggested that they expected to make some impact on health in the future, though it is recognised that the time lags, and their unpredictable nature, means a real impact on health gain can take many years to materialise and is extremely difficult to measure. Nevertheless, many of the examples described previously in which Asthma UK-funded research is making an impact on clinical policies, and on product development, are likely already, or in future, to be leading to health benefits. This is described in detail in the full report on this impact analysis but a few key examples are contained in Additional files 3, 4, and 5 which respectively describe the health gains that have arisen from the Asthma UK-funded contributions to research on leukotriene receptor antagonists [37] and on immunotherapy for allergic rhinitis, and the potential health gains from the research on peptide immunotherapy.

Health gains resulting from improved therapies are likely to have broader economic benefits in terms of reducing the working days lost through ill-health [2]. In addition, school children sitting exams during the hay fever season can suffer [38]; they might benefit from immunotherapy.

There have also been broader economic benefits to the UK from some of the cases of product development, including from the work of Tim Williams, because UK companies have been involved in undertaking some of the development. Furthermore, the two spin-out companies described above, Circassia and Synairgen, are UK based.

### A summary of impacts from each type of funding

The long-term funding for the professorial chairs has resulted in many impacts across the full range of payback categories, and the establishment of the MRC-Asthma UK Centre is a major additional benefit that can be at least partially attributed to the professorial chairs. Interview and case study evidence suggests both chairs showed considerable leadership in building up their multi-disciplinary departments that formed core elements of the Centre.

The case study approach identified the success of the MRC-Asthma UK Centre in Allergic Mechanisms of Asthma in making scientific and medical breakthroughs, training the next generation of scientists and doctors focused on asthma research, promoting collaboration, and attracting funding from other sources and increasing the funds available for asthma research. Interview evidence confirmed the documentary evidence from the House of Lords Science and Technology Committee which stated: ‘We visited a striking example of effective collaboration at the MRC-Asthma UK Centre in Allergic Mechanisms of Asthma’ [39]. Asthma UK’s strategy documents [6] help inform the Centre’s strategies. These successes and the pioneering nature of the collaborative Centre are further analysed in an additional file which summarises key points from the case study based on the Centre [see Additional file 6]. The creation of the Centre reflects current thinking on the importance of both translational health research, and the collaborations between researchers and service providers and across institutions and disciplines [40-42].

The medium-term funding for the fellowships enabled some of them to develop a strand of research that made a range of impacts. Various individual projects and fellowships provided a very small return according to the survey, but others contributed considerably according to both the surveys and the case studies.

## Discussion

As the only national charity dedicated to asthma in the UK, Asthma UK commissioned HERG/RAND Europe to apply the Payback Framework to a comprehensive analysis of the various types of research funding traditionally provided by the charity in order to shape the charity’s future research funding to maximise benefits for people with asthma. Based on the funded projects included in this research, it appears that Asthma UK’s previous research funding approaches have made some important contributions to research returns in the full range of categories. Whilst there are generally fewer impacts identified in the difficult to measure, and time lag dependent, categories such as impact on healthcare, some examples have been described. Various individual projects and fellowships provided a very small return according to the survey (and, for example the 13% of projects claiming to have made an impact on policies is much lower than the 60% figure claimed by primary studies funded in the English HTA programme, which admittedly is much more oriented to meeting the expressed needs of the NHS). Nevertheless, some Asthma UK-funded projects contributed considerably and the long-term funding of the professorial chairs has led to many and varied impacts, including in part to the establishment of the MRC-Asthma UK Centre.

The limitations of the study include the nature of the survey which attempted to be comprehensive, but some respondents thought it was too long and overall the response was less than 60%. As noted, there is a possible bias in the results as the average number of publications already known to Asthma UK prior to the surveys was slightly higher for the projects on which surveys were returned, than for those that were not. Inevitably there are also gaps in the data that can be collected, especially through surveys. A possible limitation in the other direction is that if PIs have not fully answered all the questions there could be some underestimation of the impacts, for example of the amount of follow-on funding. Furthermore, there are variations in the extent to which impacts have been demonstrated to result from the specific funding provided by Asthma UK. Whilst relying on PIs to complete surveys about their own research is clearly a potential limitation, the limited amount of evidence available from this study is in line with that from previous studies which appears to indicate that, at least in studies where there is no clear correlation between the replies given and future funding, researchers do not routinely exaggerate the impacts of their research [8]. Important impacts have been reported from the funding of professorial chairs, and also fellowships, but it is difficult to assess how much of these impacts should be attributed to Asthma UK funding.

Strengths of the study include the wide coverage of Asthma UK research, which was achieved by sending a survey to all fellows and to the PIs of projects funded and completed over a 10 year time period. In addition, the case studies provide detailed analysis of some of the research funded by the charity. Some of the case studies provide not only more detailed information than comes from the surveys, but also examples of whole categories of impact that had not been mentioned in the survey response from the project. All aspects of the data collection, analysis and detailed write-up in the full report are informed by the well-established Payback Framework which is reported to be the most widely used approach to assess the impacts from health research [8-10]. Previous reviews of studies assessing the impact from programmes of health research [8] do not seem to report a study that has combined such comprehensive coverage of the work of diverse streams of funding from one research funder with an analysis of the organisational mechanisms contributing to achieving the impacts; given the overall objective of the study, this is significant.

### Use of the findings to inform Asthma UK’s strategy

Overall this analysis has given Asthma UK a unique insight into its research and provided information to guide its future strategy. It has also shown that a medical research charity, even one with relatively modest funds, can make some significant contributions - not just in traditional areas such as knowledge production, but also in health policies, product development and improved healthcare. In particular, the analysis has highlighted to Asthma UK:

▪ the importance of offering a diverse approach to research funding to create a range of returns on investment;

▪ the merits in terms of impacts of funding up-and-coming scientists to establish them in their careers, and of providing more costly long-term support to exceptional senior research leaders who can pioneer large-scale developments in a particular research field;

▪ the niche role that charitable patient organisations have in providing key pump-priming funding to enable leading researchers to make major breakthroughs that they can then take to the larger general medical research funders for more substantial support, which is particularly important given the relatively small budget available to Asthma UK to fund research;

▪ that Asthma UK’s investment into medical research directly to improve the quality of life of people with asthma, though important, has been relatively small; and

▪ that the success of the MRC-Asthma UK Centre in Allergic Mechanisms of Asthma builds partly on Asthma UK’s series of strategy documents, starting with that from 2002 [6], which helped inform the Centre’s strategies, and the creation of the Centre reflects, and contributed to, current thinking on the importance of translational health research and how best to organise health research systems to meet the needs of patients [40-42].

The findings of HERG’s evaluation placed Asthma UK in a strong position to re-focus its efforts, define the future vision for Asthma UK research, and publish its 2011–2016 research strategy. This strategy was informed at an underpinning level by the results of HERG’s analysis in terms of the mechanisms the charity will employ to fund research. One of the key features of the new research strategy is the establishment of a second research centre focused on improving the quality of life of people with asthma, which will become known as the Asthma UK Centre for Applied Research. Asthma UK wishes to take the collaborative approach which worked so well with the MRC-Asthma UK Centre and create a similar network of leading researchers to develop large scale clinical trials and other investigative studies across the UK.

Whilst the retrospective impact analysis was completed in time to feed into the revised research strategy reported here, Asthma UK continues to monitor the progress of, and be associated with, some of the most successful developments. In the case of Synairgen the findings from the Phase II trials received significant media coverage in April 2012 because the inhaled drug (SNG001) significantly reduced asthma symptoms during the critical first week of infection and reduced the number of exacerbations. According to Stephen Holgate, quoted in the company’s press release: ‘This is a really promising breakthrough for the future treatment of asthma…. This trial is an important milestone in the development of our SNG001 programme from its origins in research supported by the MRC, Asthma UK, the British Lung Foundation, the National Institute of Health Research and the University of Southampton, to today’s exciting results in this ‘real world’ asthma study’ [43].

The progress made by some of the examples of research since the completion of the retrospective impact analysis conducted for Asthma UK illustrates that the relationship between analysing impact and revising the research funding strategy is likely to be a continuing process. The findings of such a retrospective impact analysis can, of course, be used to justify past expenditure, but as demonstrated here the retrospective analysis can also inform the strategies for organisation of health research with the aim of enhancing the level of wider impacts achieved. Some previous applications of the Payback Framework have examined the research impacts from various streams of funding from the same research funding organisation [12,44], and there is growing international interest in applying such approaches [45]. This current application of the Framework to the research funded by Asthma UK is more comprehensive in scope, and, in relation to the professorial chairs, provides for the first time, as far as the authors are aware, a detailed analysis of how long-term chair funding can successfully lead to the creation of an innovative Centre that aims to translate research into improved patient care.

## Conclusions

Research funders will continue to be interested in systematically analysing the full range of impacts from the health research they fund. Through this piece of research, we have demonstrated that adapted versions of the Payback Framework can be used to conduct an assessment of the outputs and societal impacts from a portfolio of health research in a comprehensive way and, more significantly, that these can be used not only to help justify research expenditure but also to help inform the strategy of health research funders.

We systematically show all types of Asthma UK’s research funding assessed are making impacts at different levels, with the chairs and pioneering collaborative Centre being particularly significant. Whilst inevitably only a minority of individual projects and fellowships directly contributed to societal impacts, some of the research portfolio is influencing asthma-related clinical guidelines, and some contributing to product development. The study’s findings, especially in relation to the Centre, are being used to inform research funding strategies to promote the achievement of impact.

## Endnotes

^a^The full report and volume of case studies describing the retrospective impact analysis of Asthma UK-funded research will eventually be made available on the charity’s web site.

^b^Major clinical guidelines reviewed included: British Thoracic Society/Scottish Intercollegiate Guidelines Network: British Guideline on the Management of Asthma (2008, revised 2009); BSACI guidelines for the management of allergic and non-allergic rhinitis (2008); The European Pediatric Asthma Group: Diagnosis and treatment of asthma in childhood: a PRACTALL consensus report (2008); Chronic cough due to asthma: ACCP evidence-based clinical practice guidelines (2006); Allergen immunotherapy: A practice parameter (several editions produced by the Joint Task Force on Practice Parameters representing the American Academy of Allergy, Asthma and Immunology; the American College of Allergy, Asthma and Immunology; and the Joint Council of Allergy, Asthma and Immunology); Allergic Rhinitis and its Impact on Asthma (ARIA) 2008 Update (in collaboration with the Word Health Organization, GA^2^LEN and AllerGen); Global Strategy for the Diagnosis and Management of Asthma in Children 5 Years and Younger, Global Inititative for Asthma (2009).

## Abbreviations

HERG: Health Economics Research Group; LTE4: Leukotriene E_4_; LTRAs1: Leukotriene receptor antagonists; MRC: Medical Research Council; NHS: National Health Service.

## Competing interests

The research team from the Health Economic Research Group (HERG) (Stephen Hanney (SH), Teresa Jones (TJ)) and RAND Europe (Amanda Watt (AW)) received funding for Asthma UK to conduct this study. This was a collaborative project between Asthma UK and the research team, but the research team was given independence in the key aspects of the project. Given the nature of the study project it was desirable for the research team from the Health Economics Research Group, Brunel University, and RAND Europe to work with Asthma UK on various matters. Asthma UK conceived the original project, but the research team led on all the remaining aspects, including the design of the project. Asthma UK used their access to researchers to distribute the surveys that had been designed by the research team. The case studies and all the data analysis and interpretation, and preparation of the findings were undertaken independently by the research team, with advice from Asthma UK.

Leanne Metcalf (LM) is Assistant Director, Research & Practice at Asthma UK.

## Authors’ contributions

LM conceived the project and assisted SH in designing it. SH led the data gathering assisted by AW. SH led the analysis and interpretation of the data, assisted by AW and TJ, with advice from LM. SH drafted the article, with support from LM regarding the links to Asthma UK’s strategy, and LM, AW and TJ suggested revisions. All authors approved the final version.

## Supplementary Material

Additional file 1 **Assessment of returns from research funded by Asthma UK. **Description: Survey used to gather data about Asthma UK-funded projects and fellowships. Click here for file

Additional file 2 **Evaluating the returns from research funded by Asthma UK. **Basic semi-structured schedule for case study interview with Principal Investigators that was amended to meet the circumstances of each individual interview. Click here for file

Additional file 3 **Extract from case study on the impacts from Tak Lee’s Asthma UK’s Professorial Chair funding: contribution to the development of anti-leukotriene medicines and the treatment of aspirin-sensitive asthma. **Description: This is an extract from the full case study on the range of impacts from Tak Lee’s Asthma UK’s Professorial Chair funding, and focuses on just one of the areas in which he made an important contribution - contribution to the development of anti-leukotriene medicines and the treatment of aspirin-sensitive asthma. Click here for file

Additional file 4 **Asthma UK’s project funding: summary of case study on contribution of Stephen Durham’s projects to the evidence base supporting the use of immunotherapy. **Description: This is a summary of the full case study based on Asthma UK’s project funding to support the research of Stephen Durham that made a major contribution to the evidence base supporting the use of grass-pollen immunotherapy. Click here for file

Additional file 5 **Asthma UK’s project/fellowship funding: account of pump-priming research leading to successful early trials of peptide immunotherapy and potential health gains. **Description: This is a version of the account in the main Impact analysis report to Asthma UK that describes how Asthma UK’s project/fellowship funding played a key pump-priming role in funding research that is leading to successful early trials of peptide immunotherapy that has the potential to produce health gains. Click here for file

Additional file 6 **Summary of case study on the emerging impacts from the MRC-Asthma UK Centre in Allergic Mechanisms of Asthma. **Description: This is a summary of a few of the key points from the full case study on the emerging impacts from the MRC-Asthma UK Centre in Allergic Mechanisms of Asthma. Click here for file

## References

[B1] Editorial Unknown quantities Nature 2010 465 665 666 20535155 10.1038/465665b

[B2] UK Evaluation Forum Medical research: assessing the benefits to society 2006 London: Academy of Medical Sciences

[B3] Smith R Measuring the social impact of research BMJ 2001 323 528 10.1136/bmj.323.7312.528 11546684 PMC1121118

[B4] Pang T Terry RF The PloS Medicine editors WHO/PloS collection “No health without research”: a call for papers PloS Medicine 2011 8 1

[B5] Hanney S Grant J Wooding S Buxton M Proposed methods for reviewing the outcomes of health research: the impact of funding by the UK’s ‘Arthritis Research Campaign’ Health Res Policy Syst 2004 2 4 10.1186/1478-4505-2-4 15272939 PMC503400

[B6] Lee TH Barnes J Where next in basic allergy? Clin Exp Allergy 2002 32 499 506 10.1046/j.0954-7894.2002.01367.x 11972593

[B7] Buxton M Hanney S Morris S Sundmacher L Mestre-Ferrandiz J Garau M Sussex J Grant J Ismail S Nason E Wooding S Medical research - What’s it worth? estimating the economic benefits from medical research in the UK 2008 London: UK Evaluation Forum (Academy of Medical Sciences, MRC, Wellcome Trust)

[B8] Hanney S Buxton M Green C Coulson D Raftery J An assessment of the impact of the NHS health technology assessment programme Health Technol Assess 2007 11 53 10.3310/hta11530 18031652

[B9] Canadian Academy of Medical Sciences Making an impact: a preferred framework and indicators to measure returns on investment in health research 2009 Ottowa: Canadian Academy of Medical Sciences

[B10] Banzi R Moja L Pistotti V Facchini A Liberati A Conceptual frameworks and empirical approaches used to assess the impact of health research: an overview of reviews Health Res Policy Syst 2011 9 26 10.1186/1478-4505-9-26 21702930 PMC3141787

[B11] Buxton M Hanney S How can payback from health services research be assessed? J Health Serv Res Policy 1996 1 35 43 10180843

[B12] Wooding S Hanney S Buxton M Grant J Payback arising from research funding: evaluation of the arthritis research campaign Rheumatology (Oxford) 2005 44 1145 1156 10.1093/rheumatology/keh708 16049052

[B13] Oortwijn WJ Hanney SR Ligtvoet A Hoorens S Wooding S Grant J Buxton M Bouter L Assessing the impact of health technology assessment in The Netherlands Int J Technol Assess Health Care 2008 24 259 269 18601793 10.1017/S0266462308080355

[B14] Hanney S Gonzalez-Block M Buxton M Kogan M The utilisation of health research in policy-making: concepts, examples and methods of assessment Health Res Policy Syst 2003 1 2 10.1186/1478-4505-1-2 12646071 PMC151555

[B15] Jose PJ Griffiths-Johnson DA Collins PD Walsh DT Moqbel R Totty NF Truong O Hsuan JJ Williams TJ Eotaxin: a potent eosinophil chemoattractant cytokine detected in a guinea-pig model of allergic airways inflammation J Exp Med 1994 179 881 887 10.1084/jem.179.3.881 7509365 PMC2191401

[B16] Christie PE Tagari P Ford-Hutchinson AW Charlesson S Chee P Arm JP Lee TH Urinary leukotriene-e4 concentrations increase after aspirin challenge in aspirin-sensitive asthmatic subjects Am Rev Respir Dis 1991 143 1025 1029 10.1164/ajrccm/143.5_Pt_1.1025 1850964

[B17] Durham SR Walker SM Varga EM Jacobson MR O’Brien F Noble W Till SJ Hamid QA Nouria-Aria KT Long-term clinical efficacy of grass-pollen immunotherapy N Engl J Med 1999 341 468 475 10.1056/NEJM199908123410702 10441602

[B18] Oldfield WL Larche M Kay AB Effect of T-cell peptides derived from Fel d 1 on allergic reactions and cytokine production in patients sensitive to cats: a randomised controlled trial Lancet 2002 360 47 53 10.1016/S0140-6736(02)09332-7 12114041

[B19] Asthma UK Asthma UK says farewell to world-leading professorhttp://www.asthma.org.uk/news-centre/latest-news/2012/01/asthma-uk-says-farewell-to-world-leading-professor

[B20] Thomas M McKinley RK Mellor S Watkin G Holloway E Scullion J Shaw DE Warlaw A Price D Pavord I Breathing exercises for asthma: a randomised controlled trial Thorax 2009 64 55 61 19052047 10.1136/thx.2008.100867

[B21] Bott J Blumenthal S Buxton M Ellum S Falconer C Garrod R Guidelines for the physiotherapy management of the adult, medical, spontaneously breathing patient Thorax 2009 64 Suppl 1 i1 i51 10.1136/thx.2008.110726 19406863

[B22] Scadding GK Durham SR Mirakian R Jones NS Leech SC Farooque S BSACI guidelines for the management of allergic and non-allergic rhinitis Clin Exp Allergy 2008 38 19 42 18081563 10.1111/j.1365-2222.2007.02888.x PMC7162111

[B23] Bacharier LB Boner A Carlsen KH Eigenmann PA Frischer T Gotz M Helms PJ Hunt J Liu A Papadopoulos N Platts-Mills T Pohunek P Simons FER Valovirta E Wahn U Wildhaber J The European Pediatric Asthma Group Diagnosis and treatment of asthma in childhood: a PRACTALL consensus report Allergy 2008 63 5 34 10.1111/j.1398-9995.2008.01640.x 18053013

[B24] Dicpinigaitis PV Chronic cough due to asthma: ACCP evidence-based clinical practice guidelines Chest 2006 129 1 Suppl 75S 79S 16428696 10.1378/chest.129.1_suppl.75S

[B25] Brightling CE Bradding P Symon FA Holgate ST Wardlaw AJ Pavord ID Mast-cell infiltration of airway smooth muscle in asthma N Engl J Med 2002 346 1699 1705 10.1056/NEJMoa012705 12037149

[B26] British Thoracic Society Scottish Intercollegiate Guidelines Network British guideline on the management of asthma 2008 England http://www.brit-thoracic.org.uk/Portals/0/Guidelines/AsthmaGuidelines/PreviousAsthmaGuidelines/asthma_final2008.pdf

[B27] Saglani S Nicholson AG Scallan M Balfour-Lynn I Rosenthal M Payne DN Bush A Investigation of young children with severe recurrent wheeze: any clinical benefit? Eur Respir J 2006 27 29 35 10.1183/09031936.06.00030605 16387932

[B28] Berry MA Shaw DE Green RH Brightling CE Wardlaw AJ Pavord ID The use of exhaled nitric oxide concentration to identify eosinophilic airway inflammation: an observational study in adults with asthma Clin Exp Allergy 2005 35 1175 1179 10.1111/j.1365-2222.2005.02314.x 16164444

[B29] Jadad AR Sigouin C Mohide PT Levine M Fuentes M Risk of congenital malformations associated with treatment of asthma during early pregnancy Lancet 2000 355 119 10.1016/S0140-6736(99)02542-8 10675175

[B30] Tata LJ Lewis SA McKeever TM Smith CJ Doyle P Smeeth L Gibson JE Hubbard RH Effect of maternal asthma, exacerbations and asthma medication use on congenital malformations in offspring: a UK population-based study Thorax 2008 63 981 987 10.1136/thx.2008.098244 18678701

[B31] British Thoracic Society and Scottish Intercollegiate Guidelines Network British Guideline on the Management of Asthma: a national clinical guideline. May 2008. Revised June 2009 http://www.brit-thoracic.org.uk/Portals/0/Guidelines/AsthmaGuidelines/sign101%20revised%20June%2009.pdf

[B32] Xystrakis E Kusumakar S Boswell S Peek E Urry Z Richards DF Adikibi T Pridgeon C Dallman M Loke TK Robinson DS Barrat FJ O’Garra A Lavender P Lee TH Corrigan C Hawrylowicz CM Reversing the defective induction of IL-10-secreting regulatory T cells in glucocorticoid-resistant asthma patients J Clin Invest 2006 116 146 155 16341266 10.1172/JCI21759 PMC1307558

[B33] Christie PE Smith CM Lee TH The potent and selective sulfidopeptide leukotriene antagonist, SK&F 104353, inhibits aspirin-induced asthma Am Rev Respir Dis 1991 144 957 958 10.1164/ajrccm/144.4.957 1928974

[B34] Laitinen LA Laitinen A Haahtela T Vikka V Spur BW Lee TH Leukotriene E and granulocytic infiltration into asthmatic airways The Lancet 1993 341 989 990 10.1016/0140-6736(93)91073-U 8096945

[B35] Wark PA Johnston SL Bucchieri F Powell R Puddicombe S Laza-Stanca V Holgate ST Davies DE Asthmatic bronchial epithelial cells have a deficient innate immune response to infection with rhinovirus J Exp Med 2005 201 937 947 10.1084/jem.20041901 15781584 PMC2213100

[B36] Synairgen IFNβ in Asthmahttp://www.synairgen.com/programmes/ifn-β-in-asthma.aspx23671710

[B37] O’Byrne PM Gauvreau GM Murphy DM Efficacy of leukotriene receptor antagonists and synthesis inhibitors in asthma J Allergy Clin Immunol 2009 124 397 403 10.1016/j.jaci.2009.05.029 19608262

[B38] Walker S Khan-Wasti S Fletcher M Cullinan P Harris J Sheikh A Seasonal allergic rhinitis is associated with a detrimental effect on examination performance in united kingdom teenagers: case-controlled study J Allergy Clin Immunol 2007 120 381 387 10.1016/j.jaci.2007.03.034 17560637

[B39] House of Lords Science and Technology Committee Allergy. Volume 1: Report. HL Paper 166-1 2007 London: The Stationery Office

[B40] Cooksey D A review of UK health research funding 2006 London: HM Treasury 10.1136/bmj.39059.444120.80 PMC1702444 17170394

[B41] Hanney S Kuruvilla S Soper B Mays N Who needs what from a national health research system: lessons from reforms to the English department of Health’s R&D system Health Res Policy Syst 2010 8 11 10.1186/1478-4505-8-11 20465789 PMC2881918

[B42] National Institute for Health Research Delivering health research: national institute for health research progress report 2008/9 2009 London: Department of Health

[B43] Synairgen Positive Phase II asthma clinical trial data http://www.synairgen.com/media/1536/19%20april%202012%20Phase%20II%20press%20release%20final.pdf

[B44] Nason E Curran B Hanney S Janta B Hastings G O’Driscoll M Wooding S Evaluating health research funding in Ireland: assessing the impacts of the Health Research Board of Ireland’s funding activities Res Eval 2011 20 193 200 10.3152/095820211X12941371876823

[B45] Adam P Solans-Domènech M Pons JMV Aymerich M Berra S Guillamon I Sánchez E Permanyer-Miralda G Assessment of the impact of a clinical and health services research call in Catalonia Res Eval 2012 21 319 328 10.1093/reseval/rvs024

