# Supplementary material for: Conducting retrospective impact analysis to inform a medical research charity’s funding strategies: the case of Asthma UK
Source: Allergy Asthma Clin Immunol. 2013 May 7;9(1):17. doi: 10.1186/1710-1492-9-17 (PMC3660238; doi:10.1186/1710-1492-9-17)
Supplement: Additional file 1 — Assessment of returns from research funded by Asthma UK. Description: Survey used to gather data about Asthma UK-funded projects and fellowships. [file 1710-1492-9-17-S1.pdf]

HERG
Health Economics Research Group

Assessment of returns from research funded by Asthma UK

asthma UK

Details of *research project/fellowship* (*delete as applicable when pre-completing*)

Please confirm if the following pre-completed information on this page is correct. If not, or if the information has not been fully pre-completed, please provide us with the correct information.

1	**Type of award**	Project grant / Fellowship (delete as applicable when pre-completing)
2	**Title of *research project/programme***	project if project grant; programme if fellowship (*amend as applicable when pre-completing*)
3	***Project ID/Fellowship ID***	*Delete as applicable when pre-completing*
4	***Lead researcher (grantholder)*<br>*Research Fellow***	If a project<br>If a fellowship (*amend as applicable when pre-completing*)
5	**Host institution where the *project/fellowship* was based**	*Delete as applicable when pre-completing*
6	**Details of co-applicants (names and institutions)**	
7	**Details of collaborators (names and institutions)**	
8	**Names of individuals employed through the grant**	for Fellowships, this will be the fellows themselves plus any support staff; for project grants, this will be post doctoral researchers etc
9	**Amount funded by Asthma UK**	

10	**Project duration**	**X months**	**Start date**		**End date**	
11	**Please give details of the amounts and source of any other funds used in this research:**					

**Funding organisation**	**Amount**	**Area of the project/fellowship that funds contributed to**

If you have any questions about completing the questionnaire please contact Dr Steve Hanney at HERG: 01895 265444, email: stephen.hanney@brunel.ac.uk

Thank you for your assistance.

Please return via email (researchreturns@asthma.org.uk) or post to Asthma UK in the enclosed freepost envelope by (date)

Please note:
The focus of this evaluation is the various research funds administered by Asthma UK and **NOT** the performance of individual researchers/project teams

If the person completing this questionnaire is someone other than the lead researcher, please provide your name and contact details			
Name	Post title	Telephone number	Email address

Type of research funded

In accordance with the UKCRC Health Research Classification System, please cross (X) the appropriate box to indicate which of the following best describes the research activity funded through this award

Please indicate only one type of research activity to describe the principal focus of the research

Research Activity	Description	Examples	X
Underpinning research	Research that underpins investigations into the cause, development, detection, treatment and management of asthma or associated allergy	▪ Studies of normal biology including molecular, cellular and physiological structures and function<br>▪ Development of novel underpinning research measures and analytical methodologies	
Aetiology	Identification of determinants that are involved in the cause, risk or development of asthma or associated allergy	▪ Identification and characterisation of endogenous factors known or suspected to be involved in the cause, risk or development of asthma or associated allergy<br>▪ Environmental, psychological, economic or external factors associated with the cause, risk or development of asthma or associated allergy	
Prevention of disease and conditions, and promotion of well-being	Research aimed at the primary prevention of asthma or associated allergy, or promotion of well-being	▪ Development, implementation and evaluation of interventions to modify personal or group behaviours and lifestyles affecting health and well-being<br>▪ Research on vaccines for prevention of disease	
Detection, screening and diagnosis	Discovery, development and evaluation of diagnostic, prognostic and predictive markers and technologies	▪ Discovery, development and preclinical testing of novel markers (that may be derived from patient samples) and technologies for use in detection, diagnosis, prediction, prognosis and monitoring<br>▪ Studies investigating population screening programmes	
Development of treatments and therapeutic interventions	Discovery and development of therapeutic interventions and testing in model systems and preclinical settings	▪ Identification and development of pharmaceutical small molecules, therapeutic vaccines, antibodies and hormones<br>▪ Discovery and development of medical devices	
Evaluation of treatments and therapeutic interventions	Testing and evaluation of therapeutic interventions in clinical, community or applied settings	▪ Clinical application and evaluation of pharmaceutical small molecules, therapeutic vaccines, antibodies and hormones in humans<br>▪ Application and evaluation of medical devices in humans, and all aspects of testing, evaluation and provision of complementary approaches to conventional medicine, in a clinical, community or applied setting	

Management of diseases and conditions	Research into individual care needs and management of asthma or associated allergy	▪ Studies of patients and service user care needs including quality of life, management of acute and chronic symptoms, management of side effects, long term morbidity and psychological impact of illness<br>▪ Studies into all aspects of the management of asthma and associated allergy by health and social care professionals	
Health and social care services research	Research into the provision and delivery of health and social care services, health policy and studies of research design, measurements and methodologies	▪ Examining the organisation and provision of health and social care services and evaluating factors affecting the quality of care<br>▪ Economic evaluation of health and social care interventions and delivery<br>▪ Evaluation of local, regional and national healthcare policy<br>▪ Development of research designs and novel methodologies for health care including treatment, management and health services research	

A. Previously notified publications

Please check the pre-completed information in **this Section** about the publications from your research project, which are mostly those about which you have previously notified Asthma UK. Please answer the questions about these publications and then complete the remaining Sections (B to I) using additional sheets if necessary.

A1. Please check the **previously notified publications listed** and:

1. delete any that were not, at least partially, a result of specific funding from Asthma UK
2. please use one of the following letters to categorise each publication:
   A = peer-reviewed journal article B = journal editorial C = journal letter
   D = published abstract in journal E = book F = book chapter
   G = non-peer-reviewed journal article H = published conference proceedings
   I = publicly available full report J = others (please specify)
3. for each publication mark the appropriate box about funding acknowledgement.

Previously notified publications	Category	Asthma UK funding acknowledged	
		Yes	No

B. Publications not previously listed

B1. Please list any **additional publications (not those listed in Section A)** that have resulted directly or indirectly from the research project.

Include any accepted publications that are in press but not any that are only at the submitted stage.
NB: If you have recently submitted a publication, please forward this information separately to evickers@asthma.org.uk.

For each publication please:

1. use one of the following letters to categorise it:
   A = peer-reviewed journal article   B = journal editorial   C = journal letter
   D = published abstract in journal   E = book   F = book chapter
   G = non-peer-reviewed journal article   H = published conference proceedings
   I = publicly available full report   J = others (please specify)

2. for each publication mark the appropriate box about Asthma UK funding acknowledgement.

Publications not previously listed	Category	Asthma UK funding acknowledged	
		Yes	No

C. Use of the research in the research system

C1. Has participation in the research led to additional formal qualifications for any members of the project team, whether or not they were included on the application, or is it likely to do so? **Yes** ☐ **No** ☐ **Don't know** ☐

If so, please give details.

**Type of qualifications awarded to project team members**	**Number of qualifications**		**Approximate intellectual contribution to the qualification made by your project (please cross (X) the appropriate box)**		
	**Gained**	**Expected**	**Considerable**	**Moderate**	**Small**
For example: MD	1		X		
MD					
PhD					
MSc/ MPH					
Others (please specify)					

C2. Has participation in the research directly led to career advancement for **any members of the project team**? **Yes** ☐ **No** ☐ **Don't know** ☐

If so, please give details.

**Number of team members**	**Changes in post**		**Approximate contribution to the career advancement made by your project (please cross (X) the appropriate box)**		
	**From**	**To**	**Considerable**	**Moderate**	**Small**
For example: 2	Senior Lecturer	Professor		X	

C3. Please describe any tools for future research generated by the project and indicate if they have been used. Examples include a new or significantly improved: animal model, cell line, physiological or biochemical marker, assay, model of disease, physiological outcome measure, economic model, validated patient questionnaire etc.

C4. Have the project findings, methodology or theoretical developments generated subsequent research led by **members of the original project team?**

Yes ☐ **No** ☐ **Don't know** ☐

If so, please give details of further grants and estimate the contribution of your original research project to securing these later funds.

Later funder	Amount	Approx. importance of project to securing later funding (please cross (X) appropriate box)		
		Considerable	Moderate	Small
For example:<br>Medical Research Council	£150,000			X

C5. Are you aware of any significant ways in which your project has contributed to further research **conducted by others?**

Yes ☐ **No** ☐ **Don't know** ☐

If so, please give details of further research projects and estimate the contribution of your original research project to further research projects conducted by others.

Project team	Research project title/topic	Approx. sixe of the contribution of your project (please cross (X) the appropriate box)		
		Considerable	Moderate	Small
For example:<br>HERG, Brunel University	The cost effectiveness of a new type of inhaler		X	

C6. Please give further details of any contribution to further research that you have listed above in **C4** or **C5** that you think is of particular importance.

D. Use of research project findings in health system policy/decision making

D1. Research project findings can be used for a wide range of policy/decision making at any level of the health service. This includes influencing the policies of local healthcare units, trusts, national governmental bodies, professional bodies at a local or national level, health-care bodies in other countries, and also influencing the curriculum of teaching or training courses. **[NB: a question on the impact on practice comes later].**

Have the findings from your research project **already** been used in any such ways? **Yes** ☐ **No** ☐ **Don't know** ☐

D2. Are there any reasons for expecting the findings to be used for **future** policy/decision making? **Yes** ☐ **No** ☐ **Don't know** ☐

D3. If you have replied **Yes** to either **D1 or D2** please give details of the use and/or expected use and categorise the **level(s)** at which policies/decisions were influenced and the importance or impact of the research project's findings to the adoption of the policy(ies)/decision(s).

*Use the following letters to categorise the **level(s)** influenced; then cross (X) the appropriate impact box.*

A = Policies from national governmental bodies in the UK, eg Department of Health, NICE
B = Clinical guidelines etc from professional organisations in the UK
C = Trust policy eg care pathway/protocol etc
D = Local unit policy eg care pathway/protocol agreed by a local team of professionals, a GP practice etc
E = Curriculum of teaching or training course etc
F = Healthcare bodies in other countries
G = Others (please specify)

**Details of each policy influenced and evidence to support the claims that the policy was influenced by your specific project**<br>**eg a citation; PI invited to serve on the guideline committee following a key piece of research etc**	**Level**	**Approximate degree of impact of the project on policy/decision making (please cross (X) the appropriate box)**		
		**Considerable**	**Moderate**	**Small**
For example:<br>The research is cited in the *British Guideline on the Management of Asthma*, and in *Expert Panel Report 3 (EPR3): Guidelines for the Diagnosis and Management of Asthma* Published by the US Department of Health and Human Services, NIH/NHLBI in 2007.	B,F			X

E. Use of research project findings in product development

E1. Research project findings can be used in the development of various types of products including pharmaceuticals, diagnostic tests, medical devices etc.

Have the findings from your research project **already** been used in any such ways? **Yes** ☐ **No** ☐ **Don't know** ☐

E2. Are there any reasons for expecting the findings to be used for **future** product development? **Yes** ☐ **No** ☐ **Don't know** ☐

E3. If you have replied **Yes** to either **E1 or E2** please give details of the use and/or expected use including: any patents, licences, MTAs; details of the product and the company with whom or in which the product development is taking place; the location of product development/manufacture; the stage of development which the product has reached (eg, animal tests, prototype development, clinical trial, regulatory approval, on the market, used in the NHS, etc); and the importance or impact of the research project's findings to the product development by crossing (X) the appropriate box.

**Please give details of:**<br>▪ **any patents, licences, Material Transfer Agreements (MTAs)**<br>▪ **each product & company developing it**<br>▪ **location of any product development/manufacture**<br>▪ **stage of development, eg animal testing, clinical trials etc**	**Approximate degree of impact of project on product development (please cross (X) appropriate box)**		
	**Considerable**	**Moderate**	**Small**
For example:<br>Using a licence based on the project findings, X company developed a new inhaler device in its R&D division in Kent. It is manufactured in India. It is on the market and being used in the NHS.		X	

F. Application of the research findings through practice or behaviour

F1. The findings from research can influence the practice (including confirmed current practice) or behaviour of health service staff or patients/public, either directly or through application of research-informed policies

Have the findings from your project **already** influenced practice or behaviour? **Yes** ☐ **No** ☐ **Don't know** ☐

F2. Are there any reasons for expecting the findings to influence the **future** practice or behaviour of health service staff or patients? **Yes** ☐ **No** ☐ **Don't know** ☐

F3. If you replied **Yes** to either **F1** or **F2** please give details and supporting evidence, if possible, about the influence on the practice of health service staff or the behaviour of patients and the public, and categorise the **level(s)** at which any change occurred and how important the research project findings were in influencing the practice or behaviour.

Use the following letters to categorise the ***level(s)*** *influenced; then cross (X) the appropriate influence box.*

A = National B = At least 1 NHS Trust C = Local unit
D = Patients or their families E = The general public F = Healthcare in other countries
G = Others (please specify)

**Details of influence on practice or behaviour and evidence to support claims that practice or behaviour was influenced by your specific project**<br>**eg the project was cited in a guideline and a survey suggests that recommendation has been widely adopted; at meetings in the Trust colleagues say that they are adopting this approach**	**Level**	**Approximate degree of influence of project on practice/behaviour (please cross (X) appropriate box)**		
		**Considerable**	**Moderate**	**Small**
For example:<br>The project was one of 4 main studies showing that X was the most effective test to diagnose Y and was recommended in the British Guidelines on the Management of Asthma. A recent survey indicated that this test had now been adopted in 75% of clinics in the UK. Similar surveys in Australia also show it is being adopted following guideline recommendations.	A, F		X	

G. Health/health service benefits arising from funded research

G1. Various possible health/health service benefits can arise from the application of research project findings and the uptake of new products. These benefits include: improved health; improved service delivery; cost savings; an increase in values considered desirable eg equity.
Have any such benefits **already arisen** as the result of the application of your research project findings?

Please cross (X) the appropriate box(es).

Nature of health/health service benefits	Yes	No	Don't know
Increased health or quality of life for patients, eg reduction in symptoms			
Improvements in service delivery, eg decreased referral time			
Cost reduction in the delivery of existing services			
Equity, eg better targeting and service accessibility			
Other (please specify)			

G2. Do you expect health/health service/economic benefits arising from the applications of your research project findings or uptake of products to be evident in the **future**?

Please cross (X) the appropriate box(es).

Nature of health/health service/economic benefits	Yes	No	Don't know
Increased health or quality of life for patients, eg reduction in symptoms			
Improvements in the process of service delivery, eg decreased referral time			
Cost reduction in the delivery of existing services			
Equity, eg better targeting and service accessibility			
Other (please specify)			

G3. If you replied **Yes** to either **G1** or **G2** please specify, giving any supporting evidence: the nature of the benefits; how and why the benefits have accrued; and, where relevant, approximately how many people have received the interventions etc resulting from the research.

H. Factors influencing the utilisation of research

H1. Please state approximately how many conference/workshop presentations, press conferences/briefings, interviews (newspapers, magazines etc) and/or other media presentations have been made by members of the research team based directly or indirectly on the findings of this research project.

**Dissemination activities**	**Number**
Conferences/workshops primarily for academics	
Conferences/workshops primarily for practitioners/service users	
Press conferences/briefings	
Interviews (newspapers, magazines)	
Other media presentations (TV or radio)	
Others (please specify)	

H2. Describe any of these dissemination activities (apart from direct interaction with users) that were particularly important in achieving utilisation of the research project's findings.

H3. Was interaction with potential users (policymakers, practitioners, patients etc) a factor in actual or future research utilisation?

Interaction before starting **Yes** ☐ **No** ☐ **Don't know** ☐

Interaction during the project **Yes** ☐ **No** ☐ **Don't know** ☐

Interaction after completing the project **Yes** ☐ **No** ☐ **Don't know** ☐

H4. Please describe any aspects of such interaction that were particularly important and any other factors that account for the research being utilised, or for the lack of utilisation. These could include the timeliness or quality of the research project, the research project findings being taken up by the key stakeholders etc.

I. Additional comments

I1. If you wish, please describe any other consequences of the research project not already covered.

I2. Please describe any other ways (not previously mentioned) in which funding by Asthma UK for this research project has increased the value/impact of your research project (eg facilitating networking with researchers undertaking similar studies, greater attention being received from potential users etc).

I3. Do you have any comments about the questionnaire itself?

END

Thank you for your help
