# Supplementary material for: Conducting retrospective impact analysis to inform a medical research charity’s funding strategies: the case of Asthma UK
Source: Allergy Asthma Clin Immunol. 2013 May 7;9(1):17. doi: 10.1186/1710-1492-9-17 (PMC3660238; doi:10.1186/1710-1492-9-17)
Supplement: Additional file 2 — Evaluating the returns from research funded by Asthma UK. Basic semi-structured schedule for case study interview with Principal Investigators that was amended to meet the circumstances of each individual interview. [file 1710-1492-9-17-S2.pdf]

Evaluating the returns from research funded by Asthma UK

Protocol for case study interview with Principal Investigators: outline of semi-structured interview schedule.

The precise nature of the interview schedule for each specific interview will be determined by the prior desk analysis undertaken on: analysis of the responses made in the survey; archival and documentary reviews; and possibly previous interviews on related topics.

Introduction:

The Health Economics Research Group (HERG), Brunel University and RAND Europe are being funded by Asthma UK to assess the impact of the research funded by Asthma UK across a wide range of categories. As part of this project we are building a series of case studies to examine the payback or impact from research grants that were awarded after 1996 [or other appropriate phrasing for different types of funding mode].

STAGE 0: Opportunity Identification/Research needs Assessment

1. What was the original impetus for your project? Solely scientific curiosity? The need to fill certain gaps in knowledge? Targeting of a particular disease state? Your own clinical experience?

2. How far was your identification of the research topic influenced by:
   a. Research you had done before? Funded by whom?
   b. The research of others? If so how did you hear about this research?

3. How much interaction was involved in determining your choice of research topic? With funders? With peers internationally in a specific research community? With representatives of patient or practitioner groups? Did institutional conditions such as lab space, equipment, availability of researchers affect the research proposal?

INTERFACE A: Peer review/ Project Specification, Selection and Commissioning

1. Were any changes requested during the peer review/ project selection process?

2. Was there any negotiation involving potential users (users in any sense – maybe clinicians, maybe patients, maybe other scientists) during the project specification or commissioning processes?

3. Was there any involvement of practitioners, or even policy-makers, in the process of project selection?

4. Had your research not been funded by Asthma UK, do you think any other body would have funded the research?

STAGE 1: Inputs to Research

1. *Check on cost of project that should have been identified from the desk analysis/the survey*. Were any other resources used on the project, for example where did the overhead costs come from? Was there soft or core funding?

2. What was the institutional setting (hospital, university, research institute) for the research? How good were the research facilities?

3. Who were the main researchers? What was their level of research experience and seniority at that time and in particular had they previously worked in the research area? Did they have any valuable techniques?

STAGE 2: Processes

1. Did the methods proposed prove to be appropriate? Were all of the avenues of research suggested in the proposal successful?

2. Was there any interaction with potential users of the research during the research processes?

3. What was your role as Principal Investigator (PI) in the team and the research process? Facilitator? Research director?

4. What was the role of collaborators in the research process (both academic and industrial)?

STAGE 3: Primary Outputs

1. *Confirm/Identify any publications based on the grant that has been selected and from the subsequent stream of work following the grant.*

2. Which publications do you think were most important from this project and why? *(Then reveal citation scores based on preliminary Web of Science citation records or bibliometric analysis if available)*

3. Did this work have any impact on the agenda for your subsequent research?

4. Did it make any impact on the career of any of the research team eg contribute to research training in terms of research degrees or the gaining of additional skills? Enable them to establish themselves in the field? Assist in gaining further? Helping the lead researcher to build a team of researchers?

5. Are you aware of any other researchers who have built on this work or used the methods you developed? What is the role of collaborators in this?

6. Over what type of time-scale do you think your research influenced subsequent research?

7. Did the research spawn a new area of investigation or make a major impact on the approach used in subsequent research? If so would you go so far as to say it led to a paradigm shift in understanding in your field?

8. *If the research was clinical:* were any basic researchers also involved? If so did this influence their attitude to clinical research?

9. Were any health practitioners involved in assisting with the research, and if so did it have any impact on their attitude towards implementing research findings in general?

10. Did the project play any part in making the existing stock of international knowledge more applicable/acceptable in the UK? Did the project allow work from another field to be applied to asthma or vice versa?

11. Has the research been included in any formal reviews? *In clinical science this would be a question about systematic reviews, in basic science it is a more general question*.

12. Have you had any impact outside the field of research you are working in?

INTERFACE B: Dissemination

1. Apart from publications, what attempt did you make to disseminate the findings to academic audiences? More widely? Did you work with funders or stakeholders to do this?

2. Did you use specially designed dissemination approaches to particular audiences, for example policy briefs for policymakers? What were the most effective mechanisms for this?

3. What was the role of your networks in dissemination?

4. Did you receive support from funders/employers for dissemination? What form did this take?

STAGE 4: Secondary Outputs: informing policy or product development

1. Has the research been cited directly in any clinical guideline, audit criteria or similar document from a professional body or public policymaking body at national or local level?

2. Do you know how far the research directly influenced the formulation of any policy, or the realisation that a policy was needed?

3. Has any of the subsequent research by yourself or others that built on the project been cited in any clinical guideline, audit criteria or similar document from a professional body or public policymaking body at national or local level? Do you think this might happen in future?

4. Was the research from your project taken up by industry/has it contributed to any commercial products?

5. If the research has made some impact, what are the key reasons for this? If it has failed to have an impact what are the reasons for this? What **barriers** were there to the research having an impact/being translated? What factors **facilitated** the research having an impact/being translated?

6. Has your research had an impact on teaching for clinicians?

7. Has any advisory role to government, hospitals, industry led to an impact from your research? How did this come about?

STAGE 5: Applications

1. Have the findings from the research influenced practitioners directly through them reading the articles or hearing a presentation about the research? Has it made any impact on practice through clinical guidelines or policies based either specifically on the research or on other research that built on your research? How strong an influence has it had? Has any impact been local, regional, national or international?

2. If the research has been taken up by industry, do you know what level of sales has been achieved by any product to which it contributed?

3. Do you expect any greater take-up of the findings in the future? Where?

4. Has there been an impact on practice through your own clinical work (if you have any)? What has been the knock on effect of that on other clinicians?

STAGE 6: Public Engagement

1. Depending on answers to previous questions about involvement of the public in shaping the research agenda, ask how far there has been any interaction with patients, patient groups or the wider public about the findings and their implication. Has this led to any improvement in the way patients manage their own care or interact with therapy? Or had any impact on public attitudes to medical research?

2. Did engagement with the public/patient groups lead to changes in the researchers' perceptions of these groups?

3. Has there been a change in attitudes in the research community to involvement of the public since the time when this research was conducted?

STAGE 7: Final Outcomes: Health gain and economic benefits

1. If the research has made impact on policy or practice, or on the behaviour of the public, is there any way of assessing the benefits in terms of: patient health gain? Qualitative improvements in the way the service is delivered that increase patient and/or practitioner satisfaction? Cost savings?

2. If it is possible to assess the potential benefit for one patient, approximately how many patients might be able to benefit from the improved therapy or organisation of the service?

3. If the improved therapy based on the research has resulted in a health gain, will this also result in fewer days lost from work/ decreased benefits payments/ decreased visits to secondary healthcare?

4. If the research has resulted in commercial development is anything known about the amount of employment generated, the level of import substitution, or the revenue generated for the company by the product?

Other general questions

1. Who else should we speak to about your research?

2. Are there other questions we should have asked or things that you want to talk about?

3. Are you happy for us to contact you to follow up on details arising from the case study research?

Health Economics Research Group (HERG)

Brunel University

Uxbridge

UK.
