# Supplementary material for: Conducting retrospective impact analysis to inform a medical research charity’s funding strategies: the case of Asthma UK
Source: Allergy Asthma Clin Immunol. 2013 May 7;9(1):17. doi: 10.1186/1710-1492-9-17 (PMC3660238; doi:10.1186/1710-1492-9-17)
Supplement: Additional file 3 — Extract from case study on the impacts from Tak Lee’s Asthma UK’s Professorial Chair funding: contribution to the development of anti-leukotriene medicines and the treatment of aspirin-sensitive asthma. Description: This is an extract from the full case study on the range of impacts from Tak Lee’s Asthma UK’s Professorial Chair funding, and focuses on just one of the areas in which he made an important contribution - contribution to the development of anti-leukotriene medicines and the treatment of aspirin-sensitive asthma. [file 1710-1492-9-17-S3.pdf]

Additional File 3: Extract from case study on the impacts from Tak Lee's Asthma UK's Professorial Chair funding: contribution to the development of anti-leukotriene medicines and the treatment of aspirin-sensitive asthma.

Tak Lee was appointed as Asthma UK Professorial Chair in 1988. As part of the mission for his chair he proposed a programme of human work on leukotrienes for which he was already receiving some project funding from Asthma UK. Pioneering work on leukotrienes, molecules that are important mediators of asthmatic responses, had been conducted by Professor Bengt Samuelson, who subsequently received the Nobel Prize. Significant contributions in this field were also made for many years by Professor Frank Austen at Harvard [1,2], the late Professor Priscilla Piper and Professor E. J. Corey, who also received the Nobel Prize. When Lee was a research fellow at Harvard from 1982-4 he worked on leukotrienes with some leading researchers, including Austen, and co-authored a series of well-cited articles on research conducted in Austen's lab. For example, Lee *et al.* (1985) [3] has been cited over 1,000 times.

By the mid-1980s major breakthroughs had been made in this area, but there were still some unresolved issues Lee wanted to address and, furthermore, into the 1990s pharmaceutical companies had failed to make an effective anti-leukotriene therapy. Ian Rodger from Merck later wrote that as a consequence of the early failures, 'many people began to question the putative role of leukotrienes in asthma.' [4] Lee's long-term Professorial Chair funding provided the basis for him to conduct a long-running programme of research in this field for which he also drew in continuing project funding from Asthma UK, as well as from the MRC and the Wellcome Trust. Lee's research covered various areas in which he made significant breakthroughs, in particular in relation to leukotriene E_4 (LTE_4). He and his colleagues were the first team to show the important role of leukotrienes in mediating aspirin-sensitive asthma as reported in a paper by Christie *et al.* (1991) [5], which reported on assessing urinary LTE_4 levels as a measure of global CysLT production in aspirin-sensitive patients. Later the same year, the team showed that experimental leukotriene antagonists (that were then still some way from being commercially available) can significantly protect against aspirin-induced symptoms in people with aspirin-sensitive asthma [6]. In 1993 Lee co-authored a paper - cited over 350 times - showing, in vivo, for the first time in humans that LTE_4, which is the most stable leukotriene in the body, had an inflammatory effect because it attracted leukocytes, especially eosinophils [7]. It, therefore, had implications for the potential role of drugs that were being developed because it indicated that the potential benefits from leukotriene receptor antagonists could be greater than had previously been considered.

In terms of a health gain, Lee's stream of research was the first to identify a group of patients, aspirin-sensitive asthmatics, for whom LTRAs (leukotriene receptor antagonists) are most appropriate. This was not known at the time the medicine development programme started. Furthermore, this stream of research has been used in documents and articles promoting the use of LTRAs, including an early editorial in the *BMJ* [8] and a recommendation from the Royal College of Physicians [9]. Therefore, it is appropriate to say that the work of Lee and colleagues should share, along with that of other key researchers, some of the credit for the health gain that is resulting from the use of LTRAs. There are undoubtedly some groups for whom there will be a substantial health gain from using LTRAs as opposed to other medicines. In 2009 O'Byrne *et al.* [10] argued that there are two situations in which LTRAs 'might have particular advantages over other medicines for asthma treatment.' One is for aspirin-sensitive asthma and on this topic the first reference O'Byrne *et al.* give is to the second 1991 paper by Christie *et al.*[6] In addition to those groups, very large numbers of people in countries such as the USA and Japan use LTRAs. Most commentators accept that there are health gains for them compared to not using medicine, but there are debates about the most effective treatment for general use.

References

1. Wasserman S: **Frank Austen, MD, and the biological characterisation of slow-reacting substance of anaphylaxis and its molecular identification as the sulfidopeptide leukotrienes.** *J Allergy Clin Immunol* 2006, **118:**976-978.
2. Wasserman S: **Frank Austen, MD, and Slow-reacting Substance of Anaphylaxis in the Sulfidopeptide Leukotirine Era**. *J Allergy Clin Immunol* 2006, **118:**978-980.
3. Lee TH, Hoover RL, Williams JD, Sperling RI, Ravalese J,3rd, Spur BW, et al: **Effect of dietary enrichment with eicosapentaenoic and docosahexaenoic acids on in vitro neutrophil and monocyte leukotriene generation and neutrophil function.** *N Engl J Med* 1985, **312**:1217-1224.
4. Rodger IW: **From bench to bedside. The hurdles of discovering a new leukotriene receptor antagonist.** *Am J Respir Crit Care Med* 2000, **161(2 Pt 2):**S7-S10.
5. Christie PE, Tagari P, Ford-Hutchinson AW, Charlesson S, Chee P, Arm JP, et al: **Urinary leukotriene-e4 concentrations increase after aspirin challenge in aspirin-sensitive asthmatic subjects.** *Am Rev Respir Dis* 1991, **143:**1025-1029.
6. Christie PE, Smith CM, Lee TH: **The potent and selective sulfidopeptide leukotriene antagonist, SK&F 104353, inhibits aspirin-induced asthma.** *Am Rev Respir Dis* 1991,**144:**957-958.
7. Laitinen LA, Laitinen A, Haahtela T, Vikka V, Spur BW, Lee TH: **Leukotriene E and granulocytic infiltration into asthmatic airways.** *The Lancet* 1993, **341:**989-990.
8. Sampson A, Holgate S: **Leukotriene modifiers in the treatment of asthma. Look promising across the board of asthma severity.** *BMJ* 1998, **316**:1257-1258.
9. Royal College of Physicians: **Allergy Report. The unmet need. Working Party on the provision of allergy services in the UK.** London: Royal College of Physicians; 2003.
10. O'Byrne PM, Gauvreau GM, Murphy DM: **Efficacy of leukotriene receptor antagonists and synthesis inhibitors in asthma.** *J Allergy Clin Immunol* 2009, **124**:397-403.
