# Supplementary material for: Conducting retrospective impact analysis to inform a medical research charity’s funding strategies: the case of Asthma UK
Source: Allergy Asthma Clin Immunol. 2013 May 7;9(1):17. doi: 10.1186/1710-1492-9-17 (PMC3660238; doi:10.1186/1710-1492-9-17)
Supplement: Additional file 4 — Asthma UK’s project funding: summary of case study on contribution of Stephen Durham’s projects to the evidence base supporting the use of immunotherapy. Description: This is a summary of the full case study based on Asthma UK’s project funding to support the research of Stephen Durham that made a major contribution to the evidence base supporting the use of grass-pollen immunotherapy. [file 1710-1492-9-17-S4.pdf]

Additional File 4: Asthma UK's project funding: summary of case study on contribution of Stephen Durham's projects to the evidence base supporting the use of immunotherapy.

Several papers in Prof Stephen Durham's stream of research on immunotherapy (IT), funded in part by Asthma UK, have made an important impact on many UK and international guidelines and consensus statements. In particular, the paper in the *New England Journal of Medicine* [1] has been cited over 600 times, and an accompanying editorial stated that it provided the best evidence to date that immunotherapy with a grass-pollen extract for three to four years induces prolonged clinical remission accompanied by persistent alteration in immunologic reactivity. It has been widely cited in guidelines , for example the 2008 BTS/SIGN *British Guideline on the Management of Asthma* [2] uses it as the only evidence (albeit only classified as Grade 3 evidence) to support the statement: 'Immunotherapy for allergic rhinitis has been shown to have a carry over effect after therapy has stopped.' The guideline then goes on to state: 'Immunotherapy can be considered in patients with asthma where a clinically significant allergen cannot be avoided.' Stephen Durham and Dr Samantha Walker, one of his co-authors on the *NEJM* paper, were two of the 15 co-authors of the recent guidelines produced by the Standards of Care Committee of the British Society for Allergy and Clinical Immunology (BSACI). In the guidelines entitled, *BSACI Guidelines for the Management of Allergic and Non-allergic Rhinitis* [3], several papers from Durham's stream of work are cited prominently. In relation to allergen immunotherapy Durham *et al.* (1999) [1] is one of two papers used to support the statement: 'Immunotherapy can be highly effective and it is the only treatment that is able to modify the natural history of AR [allergic rhinitis] and offer the potential for long-term disease remission.' [3] Then a paper by Walker *et al.* (2001) [4] describing an IT trial from the second half of the 1990s is cited as the only evidence to support two statements, including that allergen-specific IT has been shown to 'reduce non-specific bronchial hyper-reactivity and seasonal asthma in adults with seasonal rhino-conjunctivitis.' [3]

A team of experts nominated by both the European Academy of Allergy and Clinical Immunology and the American Academy of Allergy, Asthma and Immunology produced a guideline, *Diagnosis and Treatment of Asthma in Childhood: a PRACTALL Consensus Report,* for clinical practice in Europe and North America [5]. In the section on IT, Durham *et al.* (1999) [1] is again the only reference used to support the statement: 'The effect of immunotherapy appears to continue after treatment has stopped, resulting in prolonged clinical remission of allergic rhinitis symptoms.' [5] In the USA a series of major practice parameters or guidelines are produced by the joint task force from the various

professional bodies. Durham's stream of work is playing an increasingly important role on those guidelines, with Durham *et al.* (1999) [1] being used as a key reference, for example being just one of eight out of the 998 references in the full version of the 2008 guideline that is included in an outline that 'provides the allergist/immunologist with a referral guideline and associated rational and level of evidence.'[7].

Therefore, the work of Stephen Durham, to which Asthma UK has contributed, has played a pivotal role in supporting the case for the use of immunotherapy (IT). There has been particular resistance in the UK to using IT but Durham's stream of research has shown the efficacy of using IT for treating hay fever, and further strengthened the case for adopting it by helping to explain the mechanisms behind it, and this stream of work is continuing [6]. Through being cited so widely in guidelines, the work of Durham and his colleagues has been central to highlighting these benefits, and hence making IT more widely known as an attractive option in various circumstances. Given that Durham's research has contributed to an increased use of IT, and the use of IT provides additional health gains, and that patterns of use at least in the past were probably sub-optimal, it is reasonable to argue that any increase in IT is likely to have generated health gains. One further aspect of note here is that Durham heads a major allergy clinic at the Royal Brompton Hospital, and where appropriate recommends IT. Thus his research is directly informing his practice and resulting in health gains.

References

1. Durham SR, Walker SM, Varga EM, Jacobson MR, O'Brien F, Noble W, et al: **Long-term clinical efficacy of grass-pollen immunotherapy.** *N Engl J Med* 1999, **341:**468-475.
2. British Thoracic Society Scottish Intercollegiate Guidelines Network: **British Guideline on the Management of Asthma. England. 2008.** [http://www.brit-thoracic.org.uk/Portals/0/Guidelines/AsthmaGuidelines/PreviousAsthmaGuidelines/asthma_final2008.pdf]
3. Scadding GK, Durham SR, Mirakian R, Jones NS, Leech SC, Farooque S, et al: **BSACI guidelines for the management of allergic and non-allergic rhinitis**. *Clin Exp Allergy* 2008, **38**:19-42.
4. Walker SM, Pajno GB, Lima MT, Wilson DR, Durham SR: **Grass pollen immunotherapy for seasonal rhinitis and asthma: a randomized, controlled trial**. *J Allergy Clin Immunol* 2001, **107**:87-93.
5. Bacharier LB, Boner A, Carlsen KH, Eigenmann PA, Frischer T, Gotz M, et al: **Diagnosis and treatment of asthma in childhood: a PRACTALL consensus report.** *Allergy* 2008, **63:**5-34.
6. Shamji MH, Ljorring C, Francis JN, Calreron MA, Larché M, Kimber I, Frew AJ, Ipsen H, Lund K, Würtzen PA, Durham SR: **Functional rather than imunoreactive levels of IgG_4 correlate closely with clinical response to grass pollen immunotherapy.** *Allergy* 2012, **67:**217-226.
7. Wallace DV, Dykewicz MS. **The diagnosis and management of rhinitis: An updated practice parameter.** *J Allergy Clin Immunol* 200,**122:**S1-S84
