# Supplementary material for: Conducting retrospective impact analysis to inform a medical research charity’s funding strategies: the case of Asthma UK
Source: Allergy Asthma Clin Immunol. 2013 May 7;9(1):17. doi: 10.1186/1710-1492-9-17 (PMC3660238; doi:10.1186/1710-1492-9-17)
Supplement: Additional file 5 — Asthma UK’s project/fellowship funding: account of pump-priming research leading to successful early trials of peptide immunotherapy and potential health gains. Description: This is a version of the account in the main Impact analysis report to Asthma UK that describes how Asthma UK’s project/fellowship funding played a key pump-priming role in funding research that is leading to successful early trials of peptide immunotherapy that has the potential to produce health gains. [file 1710-1492-9-17-S5.pdf]

Additional file 5: Asthma UK's project/fellowship funding: account of pump-priming research leading to successful early trials of peptide immunotherapy and potential health gains

In the late 1990s Asthma UK funded two projects from a team at the Royal Brompton Hospital that were very important in advancing the development of peptide-based immunotherapy. Prof Barry Kay and Dr Mark Larché had some initial data, and whilst they were able to take out a patent, it needed proper project funding to take it further. They felt the best chance of obtaining such funding where the data were so potentially important, but also preliminary, came from the disease specific charity. The projects, led by Kay and Larché respectively, provided the first demonstration of asthma provoked by Major Histocompatibility Complex-restricted T cell activation, and the randomised controlled trial showed that several, short, overlapping Fel d 1 T cell peptides have potential in the treatment of cat allergy. The findings were published in a series of papers; three of the four that have been cited most often, including Oldfield *et al.* (2002) [1], acknowledge just a single funding source: Asthma UK.

Through the project funding Asthma UK played a pump-priming role for research because after the breakthroughs that came from these projects, the team were able to secure important funding from the MRC, as well as from Asthma UK for further work. The research led to the establishment of Circassia, an Imperial College London spin-out company. The company secured millions of pounds for product development, but it was important that further analysis was undertaken of the mechanisms involved. This formed an important element in Larché's Asthma UK senior fellowship from 2001-6 (T cell vaccines for asthma and allergic rhinitis). In turn, the fellowship helped Larché to leverage further funding and it also informed another Asthma UK-funded project led by Larché (Investigating the regulatory T-cell mechanisms associated with successful peptide-based immunotherapy (PIT) of cat-allergic subjects). Research tools developed during this fellowship are likely to inform further research: a two colour, fluorescent flow cytometric assay allows investigators to quantify the antigen-specific suppressive capacity of T cells [2], and a mouse model of allergic airway disease and low dose peptide immunotherapy was developed with Professor Clare Lloyd and others [3]. The funding acknowledgements for this latter paper illustrate the very wide range of funding secured to address this topic.

Kay and Larché are founding members of the Circassia which is making considerable progress towards the development of peptide vaccines for cat allergy/asthma. This, it is hoped, would improve the quality of life for patients, be a more acceptable therapy for patients and possibly reduce costs in existing services by offering shorter treatment

duration with a better safety profile. The company employs very few people directly, but is well-financed and employs a proven out-sourcing business model, working closely with a range of life science companies and service providers. A full account of the company can be seen on its website circassia.co.uk [4] and the comments below are taken from that website [accessed at 14 Oct 2012].

On 4 October 2012 the company issued a press release saying: 'Circassia Ltd, a specialty biopharmaceutical company focused on allergy, today announced the start of a pivotal phase 3 trial of its investigational ToleroMune® cat allergy treatment for cat allergen-induced rhinoconjunctivitis….The ToleroMune® cat allergy treatment phase 3 study follows positive results from a number of phase 2 trials. On September 17, the prestigious Journal of Allergy and Clinical Immunology published phase 2 data showing patients had a significantly greater reduction in symptoms compared with placebo one year after the start of the study, despite receiving only a four-dose course of treatment over 12 weeks.' [4]. This recent paper [5] represents a marks a major step forward in this continuing translation of research into improved healthcare.

References

1. Oldfield WL, Larché M, Kay AB: **Effect of T-cell peptides derived from Fel d 1 on allergic reactions and cytokine production in patients sensitive to cats: a randomised controlled trial.** *Lancet* 2002, **360:**47-53.
2. Verhoef A, Alexander C, Kay AB, , Larché M: **T cell epitope immunotherapy induces a CD4+ T cell population with regulatory activity.** *PLoS Med* 2005, Mar;2(3):e78.
3. Campbell JD, Buckland KF, McMillan SJ, Kearley J, Oldfield WL, Stern LJ, et al: **Peptide immunotherapy in allergic asthma generates IL-10-dependent immunological tolerance associated with linked epitope suppression.** *J Exp Med* 2009, **206:**1535-1547.
4. Circassia. [http://www.circassia.co.uk/]
5. Patel D, Couroux MD, Hickey P, Salapatek AM, Laidler P, Larché M, Hafner RP: **Fel d 1-derived peptide antigen desensitization shows a persistent treatment effect 1 year after the start of dosing: A randomized, placebo-controlled study.** *J Allergy Clin Immunol* 2012 online: http://dx.doi.org/10.1016/j.jaci.2012.07.028
