# Supplementary material for: Conducting retrospective impact analysis to inform a medical research charity’s funding strategies: the case of Asthma UK
Source: Allergy Asthma Clin Immunol. 2013 May 7;9(1):17. doi: 10.1186/1710-1492-9-17 (PMC3660238; doi:10.1186/1710-1492-9-17)
Supplement: Additional file 6 — Summary of case study on the emerging impacts from the MRC-Asthma UK Centre in Allergic Mechanisms of Asthma. Description: This is a summary of a few of the key points from the full case study on the emerging impacts from the MRC-Asthma UK Centre in Allergic Mechanisms of Asthma. [file 1710-1492-9-17-S6.pdf]

Additional File 6: Summary of case study on the emerging impacts from the MRC-Asthma UK Centre in Allergic Mechanisms of Asthma

The MRC-Asthma UK Centre in Allergic Mechanisms of Asthma (the 'Centre') was established in 2005 as a collaborative venture between King's College London and Imperial College London, their respective NHS partners, and both the MRC and Asthma UK [1]. Most interviewees agree it is appropriate to see the Centre as to some degree being an outcome of the years of continuing funding by Asthma UK of the two professorial chairs, Professor Tak Lee from King's College London and Professor Tim Williams of Imperial College London, who became respectively Director and Deputy Director of the Centre. The creation of the Centre was very much a pioneering venture. It is the first time an MRC Centre has been formed from two institutions, and probably only the second time the MRC combined with a medical research charity to fund a joint Centre. Analyses of research centres describe the enhanced impact that can come from long-term funding. For example, centres can facilitate a range of activities such as allowing researchers collectively to build a knowledge pool and play a key role in setting and meeting the research agendas in their field [2]. Whilst the MRC-Asthma UK Centre was only established in 2005, it was able to draw on the long-term Asthma UK funding for Lee and Williams. For example, in developing its strategies the Centre has been able to draw on the considerable experience of Lee and others in developing research strategies for Asthma UK [3].

A priority of the Centre has been to foster networking and collaboration between investigators both within, and across, the two colleges. Not only can a Centre generate a desire to work collaboratively, but also collaboration is facilitated by structural factors such as core funding, especially for facilities/infrastructure and PhD studentships. The MRC made a major contribution to the Centre in terms of additional facilities, including the protein production facility at King's and support for Professor Clare Lloyd's laboratory at Imperial; accounts of these show how they not only facilitated important science but also boosted collaboration. Securing MRC-Asthma UK Centre status has also been helpful in encouraging the institutions to provide more facilities for the Centre. Asthma UK's extra specific contribution to the Centre in the first phase was three PhD studentships – others have come from the MRC and the partner institutions. There is widespread agreement amongst interviewees that the PhD Programme created as a result of Centre funding has greatly enhanced the provision of research training in asthma at the institutions, including through the

recruitment of high standard cohorts of students, and the provision of joint supervision and support from both institutions.

The extra facilities provided by the MRC core funding and by the institutions, and the general culture of collaboration has enhanced the research environment. This is a key factor in securing increased additional research income from general health research funders. The House of Lords Science and Technology Committee referred to the striking example of effective collaboration at the Centre [4]. The Centre is also promoting translational research.

Asthma UK's core contribution to the Centre (through continuing funding of the chairs, and now the studentships) represents a major investment for the charity, but is relatively modest in terms of the Centre's overall budget. There are overlapping reasons why this investment is so important to the success of the Centre, and why, in turn, Asthma UK is getting such a large return from this investment. In various ways the MRC-Asthma UK Centre is working to ensure research findings are translated into improved care for people with asthma and training for healthcare professionals. The Centre is also benefitting from being able to draw on key elements of Asthma UK's strategy documents, and helping to ensure their implementation. Leading members of the Centre find it useful to refer to how their applications to other funding bodies address key aspects of the Asthma UK strategy documents.

The development of the Centre is benefiting from the current trends in health research funding, and in some cases has perhaps played some role in helping to advance the trends. For example, following the creation of the MRC-Asthma UK Centre another MRC Centre has been established jointly at Imperial and King's and the British Lung Foundation have started jointly funding PhDs with the MRC.

The trends with which the Centre is so compatible have been described in a series of reports such as the Cooksey Report [5] and the progress reports from the National Institute for Health Research [6]. They include: encouraging greater collaboration between researchers and service providers; encouraging greater collaboration between

researchers across institutions; and an emphasis on translational research and the greater involvement of the patient perspective. Probably the key theme behind these various trends is the aim of using research to improve patient care.

References

1. MRC-Asthma UK Centre in Allergic Mechanisms of Asthma. [http://www.asthma-allergy.ac.uk/]
2. Hanney S, Packwood T, Buxton M: **Evaluating the benefits from health R&D centres: a categorisation, a model and examples of application.** *Evaluation: the International Journal of Theory, Research and Practice* 2000, **6**:137-160.
3. Lee TH, Barnes J: **Where next in basic allergy?** *Clin Exp Allergy* 2002, **32:**499-506.
4. House of Lords Science and Technology Committee: *Allergy*. *Volume 1: Report*. *HL Paper 166-1*. London: The Stationery Office; 2007.
5. Cooksey D: *A Review of UK Health Research Funding*. London: HM Treasury; 2006.
6. National Institute for Health Research: *Delivering Health Research: National Institute for Health Research Progress Report 2008/9*. London: Department of Health; 2009.
